# Investigation of interactions between Jiuzao glutelin with resveratrol, quercetin, curcumin, and azelaic and potential improvement on physicochemical properties and antioxidant activities

**DOI:** 10.1016/j.fochx.2024.101378

**Published:** 2024-04-10

**Authors:** Yunsong Jiang, Yuxin Qin, Jayani Chandrapala, Mahsa Majzoobi, Charles Brennan, Jinyuan Sun, Xin-An Zeng, Baoguo Sun

**Affiliations:** a Key Laboratory of Geriatric Nutrition and Health, Beijing Technology and Business University, Ministry of Education, 100048, People's Republic of China; b School of Food Science and Engineering, South China University of Technology, Guangzhou 510641, People's Republic of China; c Biosciences and Food Technology, RMIT University, Bundoora West Campus, Plenty Road, Melbourne, VIC 3083, Australia

**Keywords:** Jiuzao glutelin, Functional components, Reinforcing effects, Stability improvement, Antioxidant ability

## Abstract

The interactions among small molecular functional components (FCTs) within a food matrix have become a focal point for enhancing their stability and bioactivities. Jiuzao glutelin (JG) is a mixed plant protein within Jiuzao (a protein-rich baijiu distillation by-product). This study aimed to explore the interactions between JG and selected FCTs, including resveratrol (RES), quercetin (QUE), curcumin (CUR), and azelaic acid (AZA), and the consequential impact on stability and antioxidant activity of the complexes. The findings conclusively demonstrated that the interactions between JG and the FCTs significantly enhanced the storage stability of the complexes. Moreover, the antioxidant activity of the complexes exhibited improvement compared to their individual counterparts. This study underscores the notion that JG and FCTs mutually reinforce, exerting positive effects on stability and antioxidant activity. This symbiotic relationship can be strategically employed to augment the quality of proteins and enhance the functional properties of bioactive components through these interactions.

## Highlights

• Jiuzao glutelin (JG) were combined with functional components (FCTs).
• JG structural changes after interaction were investigated.
• JG stabilities were improved after combination with FCTs.
• The antioxidant activities of the conjugates were enhanced after combination.

## 1 Introduction

Resveratrol (RES), curcumin (CUR), quercetin (QUE), and azelaic acid (AZA) are functional components (FCTs) possessing a range of functional properties, encompassing antioxidant, anti-inflammatory, anti-cancer, immune regulation, and antibacterial effects (Hoti et al., 2022). These properties render them highly valuable in healthcare products and pharmaceuticals industries. However, the susceptibility of FCTs to degradation from exposure to light, heat, and oxidation significantly hampers their potential applications. The interactions of FCTs with food mixtures can enhance their stability and introduce new properties. Complex stability can be improved by selecting appropriate packaging and storage conditions, incorporating protective agents, and utilizing composite formulations, thereby contributing to advancements in functional food development, drug design, disease treatment, and chemical biology research. (Garavand et al., 2022).

Interactions among various FCTs with food matrix biomacromolecules emerge as an exceptionally effective strategy for bolstering the stability of bioactive ingredients, controlling their release, enhancing bioavailability, and facilitating greater flexibility in formulation (Jiang et al., 2019). Interactions between proteins or polysaccharides and FTCs offer numerous advantages for the development and application of functional products (Pateiro et al., 2021). Binding proteins, peptides, and polysaccharides to FCTs can enhance their stability and prevent the active component's dissolution, precipitation, or deactivation through hydrophobic and electrostatic interactions (Pateiro et al., 2021). This process results in the encasement of FCT sites within the proteins, leading to steric hindrance effects. The protective coating shields FCTs from external factors such as oxygen, light, or other detrimental conditions that could trigger degradation or oxidation reaction (Zehra, Mobin, Aslam, & Bhat, 2023). Moreover, the structure and stability of the protein provide a protective mechanism against degradation or deactivation of the active ingredient. Furthermore, active ingredients can reciprocally enhance the stability and activities of proteins due to the existence of a synergistic effect (Jiang et al., 2019).

Jiuzao is the solid material remaining from the brewing process, primarily comprises grain residue (such as sorghum, rice, maize, and wheat), yeast, and various microorganisms (Wang et al., 2023). It is a rich source of plant protein, with a content typically between 25 and 30%, making it a pivotal resource for protein extraction (Jiang et al., 2021). Analogous to other brewing process by-products, such as spent brewers' grain (Anisha et al., 2023), Jiuzao glutelin (JG) constitutes a mixed plant protein, representing a substantial portion (20–30% of Jiuzao protein) (Jiang et al., 2020). JG encompasses essential amino acids, including lysine, isoleucine, and phenylalanine (Jiang et al., 2020) and possesses emulsification properties suitable for stabilizing, emulsifying, thickening, and other functions in food processing (Jiang et al., 2022a; Jiang et al., 2022b; Jiang et al., 2022c). Furthermore, it exhibits a gel-forming ability, which can be harnessed to prepare meat products, bread, biscuits, and other food, to improve texture and taste. The extraction and utilization of JG offer an effective means of recycling by-products from the brewing process, contributing to the waste and environmental pollution reduction (Zhu et al., 2021) akin to other spent grain materials generated by brewing processes (Lopes, Klosowski, Carvalho, & Olivato, 2022). JG demonstrates stability in the face of high temperatures, varying pH conditions, and UV radiation environment (Jiang et al., 2022a; Jiang et al., 2022b; Jiang et al., 2022c). Additionally, JG possesses antioxidant attributes, including radial scavenging and ferrous reducing abilities (FRA) (Jiang et al., 2022a). Consequently, this study leveraged interactions with JG as a strategy to enhance the stability of FCTs.

The current study explores the combination of JG with RES, QUE, CUR, and AZA to assess the effect of FCTs on the JG secondary structure and stability during storage. Additionally, changes in antioxidant properties after interaction were also evaluated by *in vitro* antioxidant activity assays. This study endeavours to provide novel combinations between FCTs and JG, yielding complexes with enhanced stability and antioxidant properties for potential use in the functional food industry.

## 2 Materials and methods

### 2.1 Materials and reagents

Jiuzao of sesame-flavour type (composed of sorghum, wheat, corn, rice, sticky rice, millet, and rice husk) was provided by Bandaojing Liquor Co., Ltd. (Zibo, China). RES, QUE, CUR, (purity >98%), and dimethylsulfoxide (DMSO) were purchased from Maclin Biochemical Co., Ltd. (Shanghai, China). Dithiothreitol (DTT), AZA (purity >95%), sodium dodecyl sulfate (SDS), and BCA assay kit were obtained from Beijing Solarbio Science & Technology Co., Ltd. (Beijing, China). The 2,2′-azino-bis (3-ethylbenzothiazoline-6-sulfonic acid (ABTS) kit was bought from Nanjing Jiancheng Institute of Biotechnology (Nanjing, China). The 2,2-diphenylpicrylhydrazyl (DPPH), FRA, and hydroxyl assay kits were purchased from Shanghai HuicH Biotech Co., Ltd. (Shanghai, China). PBS buffer was purchased from Yuanye Co., ltd (Shanghai, China). Fluorescein isothiocyanate (FITC), 5,5’-Dithio bis-(2-nitrobenzoic acid) (DTNB), 8-anilino-1-naphthalenesulfonic acid (ANS), NaCl, NaHCO_3_, and Na_2_CO_3_ were bought from Macklin Biochemical Co., ltd (Shanghai, China). EDTA Na_2_ was obtained from Beijing Dingsheng Biotechnology Co., Ltd. (Beijing, China). All other chemicals and solvents not mentioned in this section were of analytical reagent grade and obtained from Macklin Biochemical Co., ltd (Shanghai, China).

The extraction and preparation methods for JG were followed as previously described (Jiang et al., 2022a). A pulse electric filed was select as the subsidiary method to extract JG. JG was extracted using a 0.125 M of NaOH solution containing 0.05% dithiothreitol and 0.05% sodium dodecyl sulfate at 30 °C for 24 h. Finally, dialysis (Mw cutoff <1 kDa) was used to remove low-molecular salts and amino acids.

### 2.2 Interaction of JG with RES, QUE, CUR, and AZA

JG powder (mainly composed of sorghum glutelin) (Jiang et al., 2022a) obtained by freeze-dying (at −80 °C for 48 h) (Christ, Alpha 1–4 LSCbasic, Osterode, German) was dissolved in ultrapure water (adjusted to pH 8.5 with 1 M NaOH solution) to a final concentration of 1 mg/mL and magnetically stirred for 3 h at room temperature until fully dissolved. RES, QUE, and CUR were dissolved in DMSO, while AZA was dissolved in a 1 M of NaOH solution to achieve a series of solutions at room temperature. Then, 2 mL of RES, CUE, CUR (final concentrations were 20, 30, 40, 50, 60, 70, 80, 160, 240, 320, 480, 640, 800, and 960 μM), and AZA solutions (final concentrations were 80, 160, 320, 480, 640, 800, and 960 μM) were added and mixed with the same volume of JG solutions. The resulting mixtures were stirred for 4 h at room temperature in a dark environment to yield RES-JG, QUE-JG, CUR-JG, and AZA-JG solutions. Unbound FCTs were removed by dialysis for 24 h (Mw cutoff <1 kDa).

### 2.3 Characterizations of the interaction

#### 2.3.1 Scanning electron microscopy (SEM)

RES-JG, QUE-JG, CUR-JG, and AZA-JG solutions were freeze-dried at −80 °C for 48 h to obtain the powders. The morphology of the powders was evaluated by SEM (FEI Quanta 250 FEG, EFI Inc., OR, USA). The powders were affixed to a conductive paste. After gold spraying, the apparent morphology of the powders was recorded at 30 kV (Jiang et al., 2022a).

#### 2.3.2 Ultraviolet (UV) measurements

RES-JG, QUE-JG, CUR-JG (RES, QUE, and CUR concentrations are 20, 30, 40, 50, 60, 70, and 80 μM) and AZA-JG (AZA concentrations are 80, 160, 320, 480, 640, 800, and 960 μM) solutions were diluted fivefold for UV analysis. A blank sample consisting of JG solution at a concentration of 1 mg/mL and FCTs solutions were used as the references. Spectra were recorded by a UV-VIS spectrophotometer UV-2700 (Shimadzu, Japan) within a wavelength range of 240 to 320 nm (Jiang et al., 2022a).

#### 2.3.3 Fourier transform infrared (FT-IR) spectrum observation

Two grams of the freeze-dried RES-JG, QUE-JG, CUR-JG (RES, QUE, and CUR concentrations are 80, 160, and 240 μM), and AZA-JG (AZA concentrations are 320, 640, and 960 μM) powders were directly measured by a vector 33 IR spectrophotometer (Bruck, Ettingen, Germany) in the 800 to 4000 cm^−1^ range. The analysis was conducted with 32 scans at a resolution of 2 cm^−1^. JG and standard FCTs were regarded as controls.

#### 2.3.4 Circular dichroism (CD) spectrum

CD spectrum for RES-JG, QUE-JG, CUR-JG (RES, QUE, and CUR concentrations are 80, 160, and 240 μM), and AZA-JG (AZA concentrations are 320, 640, and 960 μM) solutions were collected by a J-815CD Spectrometer (JASCO, Tokyo, Japan) with a wavelength range of 195 to 300 nm equipped with 0.2-mm quartz cuvettes. Then, the scan was performed at 50 nm/min, and ten scans were averaged to enhance the accuracy of the measurements. The secondary structure of the FCTs-JG was calculated by Dicroprot software according to the CD spectrum. A 1 mg/mL of JG solution and JG mixed with the above concentrations of FCTs solutions without interaction were used as controls.

### 2.4 Fluorescence (FL)

The FL measurement methods were conducted as described by (Jiang et al., 2022c). Briefly, 50 mg of JG was mixed with one mg of FITC in 20 mL of ultrapure water with 0.15 M NaCl and NaHCO_3_-Na_2_CO_3_ (*v*/v, 9:1). The solution was thoroughly mixed and stirred at 4 °C overnight in the dark, followed by 24-h dialysis using a 100 Da membrane. The JG solution (1 mg/mL) containing fluorescein was then combined with various concentrations of RES, QUE, CUR, and AZA (0, 80, 160, 320, 480, 640, 800, and 960 μM). Changes in fluorescence were recorded at 30, 40, and 50 °C using a temperature constant incubator and a fluorescence spectrophotometer (F-4600, Hitachi, Tokyo, Japan). The fluorescence spectra of the solutions were measured at an excitation wavelength of 390 nm and an emission wavelength range of 490–550 nm. The degree of quenching was calculated using the classic or modified Stern-Volmer equations:

(1) $$\frac{F_{0}}{F}=1+K_{\mathrm{SV}}\left[C_{Q}\right]=1+K_{q}\tau_{0}\left[C_{Q}\right]$$

(2) $$\frac{F_{0}}{F}=e^{K_{\mathrm{SV}}\left[C_{Q}\right]}$$

where F_0_ and F represent the fluorescence intensity of the fluorophore in the absence and presence of a quencher; [C_Q_] is the quencher concentration; and K_SV_ is the Stern-Volmer quenching constant. K_SV_ = K_q_τ0, where K_q_ is the quenching rate constant of biomolecules; and τ0 is the average lifetime of the molecule without quencher (10^−8^ s). Eq. (1) is used to draw the change curve of the fluorophore $\frac{F_{0}}{F}$ with ligand concentration to analyse the fluorescence emission result, and Eq. (2) is used to analyse the upward Stern-Volmer curve.

RES, QUE, CUR, and AZA interacted with JG binding sites independently. The interaction among free and combined JG molecules was calculated by the following equation:

(3) $$\log\left(\frac{F_{0}-F}{F}\right)={\text{logK}}_{A}+\text{nlog}\left[C_{Q}\right]$$

where K_A_ is a binding constant that reflects the degree of reaction of JG with RES, QUE, CUR, and AZA. The value of n represents the number of possible binding sites in JG. The values of K_A_ and n can be calculated from the intercept and the slope of the $\log\left(\frac{F_{0}-F}{F}\right)$ *vs.* $\log\left[C_{Q}\right]$ plot (Fig. 3c).

The thermodynamic parameters of ΔH, ΔS, and ΔG were evaluated by the following equations:

(4) $$\ln K_{A}=-\frac{\mathrm{\Delta H}}{\mathrm{RT}}+\frac{\mathrm{\Delta S}}{R}$$

(5) ΔG=ΔH−TΔS

where ΔH, ΔS, and ΔG are the enthalpy, entropy, and free enthalpy changes, respectively, and K_A_ and R are the binding and universal gas constant (8.314 J/mol·K).

### 2.5 Measurement of sulfhydryl content change on JG surface

The sulfhydryl content change on the JG surface was quantified by dissolving the JG sample in neutral PBS to attain a 2.5 mg/mL concentration. A reaction solution was prepared by mixing 10 mM of DTNB, 86 mM Tris-HCl, 90 mM glycine, and 4 mM EDTA Na_2_. The JG-FCTs solutions (200 μL) were mixed with 20 μL of the reaction solution and incubated in the dark for 15 min at 25 °C. The absorbance was measured at 412 nm, using the JG solution as the blank. The percentage of sulfhydryl content change was calculated according to the following formula:

(6) $$\text{Sulfhydryl content change}\;\left(\%\right)=\left(\frac{S1-S0}{S0}\right)\times 100\%$$

where S_1_ is the absorbance of the samples and S_0_ is the absorbance of untreated JG.

### 2.6 Measurement of surface hydrophobicity change on JG

FCTs-JG surface hydrophobicity (H_0_) was assessed by measuring the interaction with the hydrophobic fluorescent dye of ANS, following a method with slight modifications by (Jiang et al., 2022c). Specifically, 20 μL of an 8 mM ANS solution was added to 200 μL of 0.01, 0.05, 0.1, 0.15, and 0.2 mg/mL sample solutions, prepared in neutral PBS (10 mM). FL intensity was recorded at 390 nm (excitation) and 470 nm (emission) using a Shimadzu fluorescence spectrophotometer (Tokyo, Japan). The initial slope of fluorescence intensity *versus* protein concentration plot was utilized as the index of H_0_.

### 2.7 Antioxidant abilities

ABTS, DPPH, hydroxyl radical scavenging abilities, and FRA were conducted in compliance with the instructions provided by the assay kit manufacturers (as mentioned in Section 2.1) for the RES-JG, QUE-JG, CUR-JG, and AZA-JG solutions (Jiang et al., 2022a).

### 2.8 Storage stability analysis

RES-JG, QUE-JG, CUR-JG, and AZA-JG solutions were stored in hermetically sealed containers at 4 °C, shielded from light, for a duration of five weeks (Zhang, Li, & Jiang, 2020). JG concentrations were assessed on a weekly basis. The stability of the solutions was quantified by monitoring JG content changes using a BCA assay kit. JG and JG-FCTs mixtures without interaction were regarded as control groups.

### 2.9 Statistical analysis

All the measurements were performed on at least four replicates. Data were reported as mean ± standard deviation (SD) and assessed by one-way analysis of variance (ANOVA) using SPSS 25.0 statistical program (IBM Inc., NY, USA). *p* < 0.05 was considered statistically significant.

## 3 Results and discussion

### 3.1 SEM observation

**Fig. 1:**
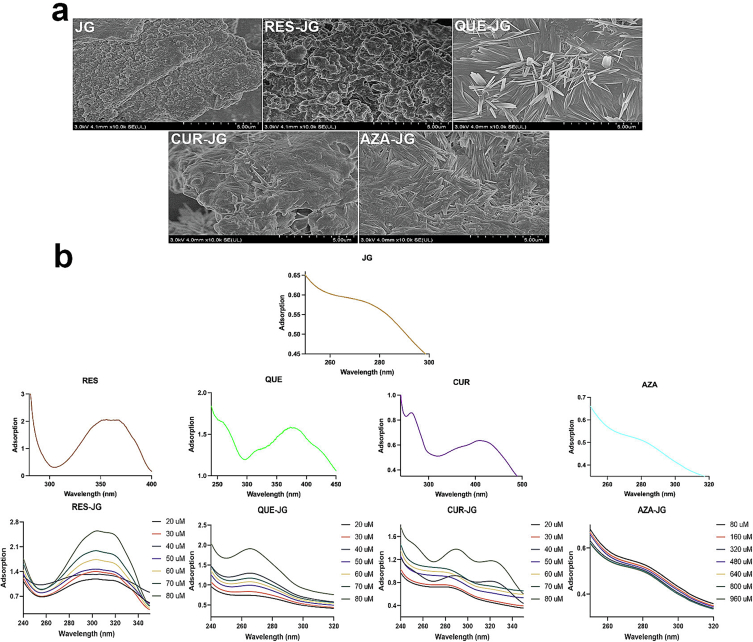
(a) Morphology of JG and JG after interactions. (b) UV spectrum of JG, JG and FCTs mixtures, and JG after interactions.

The morphology of JG displayed a blocky structure (Fig. 1a) with a smooth lump and a compact texture (Jiang et al., 2022a). However, upon reaction with RES, QUE, CUR, and AZA, notable changes were observed in JG's morphology. Specifically, QUE and AZA transformed JG's morphology from a blocky structure to a dendritic fibrous structure. This transformation is attributed to the presence of QUE and AZA on the surface of JG, leading to the manifestation of crystal textures. Conversely, there was no apparent morphological change in the combination of RES-JG and CUR-JG, suggesting that RES and CUR primarily entered the interior of JG and were seldom found on its surface. This result aligns with Zhang's investigation of pea protein, where RES, QUE, and CUR encapsulation resulted in a spherical morphology, indicating the successful encapsulation of these three compounds within the pea protein isolate (Chen, Wang, Feng, Jiang, & Miao, 2019).

### 3.2 UV analysis

JG exhibits adsorption at 280 nm (Fig. 1b) due to the presence of benzene, indole, and phenol rings in phenylalanine, tyrosine, and tryptophan, as well as n → π* transition of carbonyl, amide bonds, and aromatic amino acid residues (Jiang et al., 2022a). RES, QUE, and CUR display adsorptions at 366 nm, 245 and 374 nm, and 280 as well as 420 nm, respectively. Notably, upon interaction with RES, QUE, and CUR, the UV spectrum of JG underwent notable changes (Fig. 1b). A red shift in the peak at 280 nm was observed for RES-JG and CUR-JG, attributed to structural changes in JG (*e.g.*, α-helix, β-sheet, β- reversal, and random structure) due to steric hindrance, cis-trans isomerism, trans-ring effect, electron cloud distribution, and resonance structures within the molecules (Jiang et al., 2022c). Additionally, the addition of RES and CUR enhanced the polarity of JG, altering the pH of the solution (pH changed from 8.5 to 6.2 after interactions) (Srivastava & Alam, 2018). CUR-JG exhibited a peak at 320 nm, indicating the formation of new conjugates with different structures and electronic states, influenced by pH changes in the AZA solution affecting the secondary and tertiary structures of JG through protonation or deprotonation of amino acid residues (Tan, Ye, & Xie, 2021). pH changes resulted in protonation or deprotonation of amino acid residues, affecting JG's secondary and tertiary structures. Conversely, there was a blue shift in the spectrum of QUE-JG. QUE had a low absorption peak at 245 nm caused by the σ-σ* transition in its structure, underwent an n-σ* transition when combined with JG, resulting in a redshifted absorption (Li et al., 2022). The adsorption increased with the concentrations of FCTs in each combination, reflecting increased light path length, adsorption coefficient, and interparticle interactions. This phenomenon suggests weaker molecule aggregation improved dispersion and enhanced solubility compared to the native JG (Liu et al., 2020).

### 3.3 FT-IR analysis

**Fig. 2:**
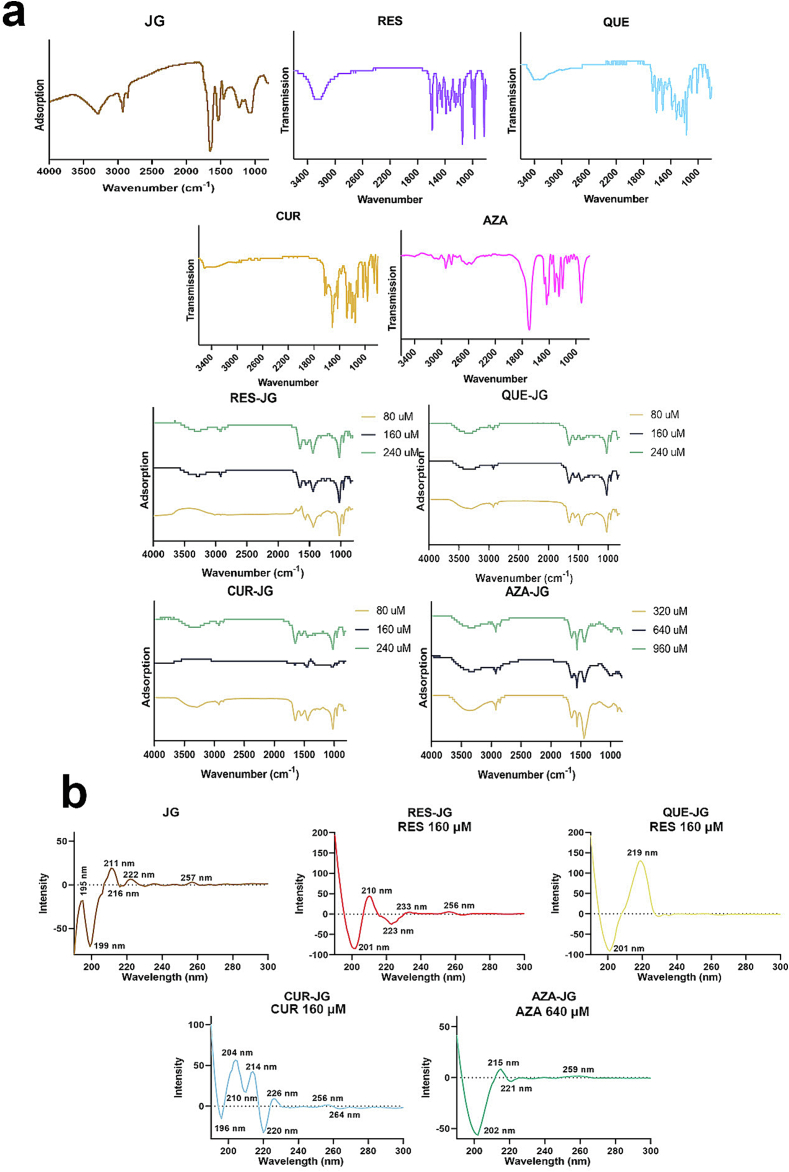
(a) FT-IR spectrum of JG, JG and FCTs mixtures, and JG after interactions. (b) CD spectrum of JG and combination with RES, QUE, CUR, and AZA.

Proteins exhibit distinctive absorption peaks in the FT-IR spectrum, including amide bands I, II, and III (1600–1700 cm^−1^, 1500–1600 cm^−1^, and 1200–1350 cm^−1^) (Jiang et al., 2022a). As depicted in Fig. 2a, JG demonstrated the characteristic FT-IR spectrum as reported previously (Jiang et al., 2022a). RES (1572.10, 1492.30, 1423.90, 1366.90, 1304.10, 1233.30, 1127.90, 947.71, and 809.41 cm^−1^) QUE (1651.30, 1591.90, 1496.10, 1430.80, 1359.00, 1298.60, 1229.60, 1183.30, 1147.70, and 986.85 cm^−1^), CUR, and AZA (2918.40, 2813.30, 1672.20, 1417.00, 1294.60, 1234.60, 1178.00, and 899.74 cm^−1^) displayed characteristic peaks in their FT-IR absorption spectrum as well. However, when JG interacted with FCTs, the adsorption pattern changed. Notably, red shifts were observed at peaks of 1028.84, 1083.32, 1455.99, 1651.25 (except RES-JG), along with a peak at 2923.56 cm^−1^ in FCTs-JG. These shifts are attributable to the increased JG solubility and decreased particle size after interaction with FCTs, resulting in a reduced particle size (Tan et al., 2021). The alteration in particle size impacts the absorption band, leading to a blue shift and inducing changes in the energy band structure due to increased internal stress within the particles (Liu et al., 2020). Furthermore, electron transitions from lower to higher energy levels can cause increased electron wave function overlap, narrowing of the band gap and energy level spacing, and a red shift in the optical absorption band and absorption edge (Tian et al., 2020). Blue shifts at 1531.68 cm^−1^ in four combinations and 1651.25 cm^−1^ in RES-JG can be attributed to the widening of the energy gap as the particle size decreases and the increase in the width between the energy level of the electron-occupied molecular orbital and the energy of the unoccupied molecular orbital (Liu et al., 2020). Significant surface tension induces lattice distortion, a decrease in lattice constant, and an increase in bond length shortenings, which further elevates the intrinsic vibration frequency of the particles' bonds, leading to a blue absorption shift (Ahmad, 2022).

### 3.4 CD analysis

Proteins exhibit distinct peaks in the CD spectrum, rendering CD spectral analysis a prevalent method for assessing protein structure. The CD region of proteins primarily encompasses the 190–300 nm range, with peptide chains constituting the main chromophores (Jiang et al., 2022a). The α-helical conformation in the CD region shows a positive peak at 190 nm and negative peaks at 208 and 222 nm. β-folding is characterized by a strong positive peak at 195–198 nm and a negative peak at 217–218 nm. The random curly conformation displays a negative peak near 198 nm and a small but broad positive peak near 220 nm (Jiang et al., 2022a). Disulfide bonds are signified by a positive peak at 255–260 nm. As depicted in Fig. 2b, JG exhibited peaks at 195 (β-folding), 199 (random curly conformation), 211 (β-bend), 216 (β-folding), 222 (random curly conformation), and 257 nm (disulfide bond). The complexes of JG with FCTs without interaction showed the same CD spectrum of JG (data not shown), indicating no change JG structure. However, after combination with FCTs, the CD peaks of JG changed. There were only peaks at 201 (random curly conformation), 210 (β-bend), 223 (α-helical), and 256 nm (disulfide bond) left in the RES-JG CD spectrum; 201 (random curly conformation) and 219 nm (random curly conformation) in QUE-JG; and 202 (random curly conformation), 215 (β-folding), 221 (random curly conformation), and 259 nm (disulfide bond) in AZA-JG. CUR changed the JG secondary structure, mainly with negative peaks at 196 (random curly conformation), 210 (β-bend), and 220 nm (α-helical) and positive peaks at 204 (β-folding), 214 (β-bend), 226 (random curly conformation), and 256 nm (disulfide bond). However, the concentrations of FCTs did not influence the characteristic peaks of JG. The peak movement of proteins can be influenced by various factors, such as changes in protein structure, pH, solvent, temperature, and protein-ligand interactions (Haque, Kaur, Islam, & Hassan, 2022). The addition of FCTs alters the charge state of the protein, leading to changes in JG's structure and conformation, as evidenced by the shift in CD peaks, corroborating the FT-IR results that the structure of JG is changed after combination with FCTs. A similar observation was reported in Zhu's study, where combining Kaffrin with ferulic acid and tetramethyl pyrazine induced changes in the secondary structure of the protein (Zhu et al., 2021).

### 3.5 FL analysis

**Fig. 3:**
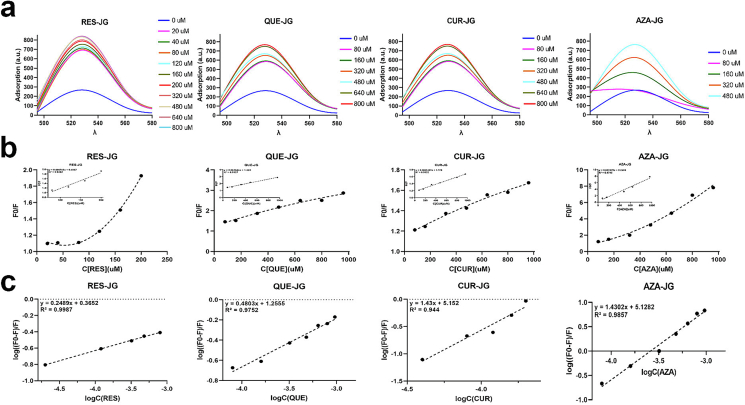
(a) FL spectrum of JG and combination with different concentrations of RES, QUE, CUR, and AZA. (b outer part) The Stern-Volmer plot of JG-FCTs system and (b inner part) the Stern-Volmer linear plot of RES, QUE, CUR, and AZA with JG. (c) The plot of Eq. (3) of RES, QUE, CUR, and AZA with JG.

Fluorescence spectroscopy is a widely adopted technique for investigating protein structure and properties. To render JG fluoresce, it was combined with FITC dye. Protein structure and conformation information can be obtained by measuring the fluorescence spectrum of protein markers. These spectrum offer insights into the internal structure, conformational state, fluorescence intensity, and fluorescence lifetime (Zhu et al., 2021). Fig. 3a shows the FL emission spectra of FITC-labelled JG, RES-JG, QUE-JG, CUR-JG, and AZA-JG at room temperature. The maximum emission wavelength for FITC-marked JG was determined to be 521.8 nm with an excitation wavelength of 390 nm. The FL intensity of FITC-labelled JG increased with higher contents of the four FCTs. Red shifts in the FL peak appeared from 522.2 to 523.8 nm, attributed to the addition of RES, QUE, and CUR leading to conformational changes in JG that cause the redshift of fluorescence emission peaks (Khan et al., 2021). When a protein undergoes a transition from a tightly folded conformation, the environment surrounding the fluorophore changes, resulting in a longer wavelength emission peak. Fluorescent dyes or labelled proteins typically interact with surrounding amino acid residues. When the conformation of the protein changes or interacts with other molecules, the fluorescent dye's environment also changes, affecting the location of the emission peak (Zhu et al., 2021). Fluorescent dyes commonly contain various functional groups, such as hydroxyl and amine, which can form hydrogen bonds or engage in charge interactions with amino acid residues in proteins. Alterations in these interactions lead to shifts in the fluorescence emission peak. Interestingly, the AZA-JG FL spectrum exhibited a blue shift, which became more pronounced with higher AZA concentrations. This result is ascribed to AZA altering the pH and polarity of the solution. The non-polar solvent reduces the interaction between the fluorescent dye and the polar molecules in the solvent, resulting in a change in the fluorophore's environment and a blue shift in the emission peak. This finding aligns with a previous study that observed spectral shifts in human serum albumin when combined with methylglyoxal, indicating conformational changes in the protein. (Khan et al., 2021).

Stern-Volmer plots are commonly used in fluorescence spectroscopy to analyse the quenching behaviour of fluorophores (Zhu et al., 2021). These plots provide valuable insights into the mechanism and kinetics of fluorescence quenching by different quenchers. Fig. 3b illustrates the Stern-Volmer plots. Among all concentrations, QUE-, CUR-, and AZA-JG exhibited a nearly linear trend (both inner and outer parts), which indicated a dynamic (collisional) quenching process. However, the Stern-Volmer plot of RES-JG exhibited a non-linear trend (the outer part) when the concentrations of RES were up to 200 μM, suggesting a complex mechanism involving static or other types of quenching (such as non-collisional mechanism) (Zhu et al., 2021).

As indicated in Table S1, the K_q_ value exceeded the highest dynamic scattering collision rate constant (2 × 10^10^ M^−1^ S^−1^) by an order of magnitude at 30, 40, and 50 °C. This observation suggests that the fluorescence quenching is primarily initiated by static quenching. Additionally, the K_SV_ value presented a trend of initial increase followed by a decrease in RES-JG, QUE-JG, and AZA-JG. While a decreasing trend was observed in CUR-JG, indicating dynamic quenching.

Temperature also influences the development of the combination (Jiang et al., 2022c). Several forces are involved, including electrostatic interaction, hydrogen bonding, hydrophobic interaction, van der Waals force, and hydrophobic repulsion in the binding process of FCTs with JG (Zhu et al., 2021). The thermodynamic parameters △H, △S, and △G were evaluated to ascertain the energy changes during these interactions. Apart from RES-JG, △G values were negative at all three examined temperatures, implying the spontaneous nature of these combinations. Conversely, the combination of RES-JG necessitated extra energy input. The influences on △H, △S, and △G stem from changes in chemical bonds during reactions. The formation of new bonds typically releases energy, resulting in a negative △H (exothermic reaction) (Zhu et al., 2021). In contrast, breaking bonds necessitate an energy absorption, leading to a positive △H (endothermic reaction).

### 3.6 Sulfhydryl content

**Fig. 4:**
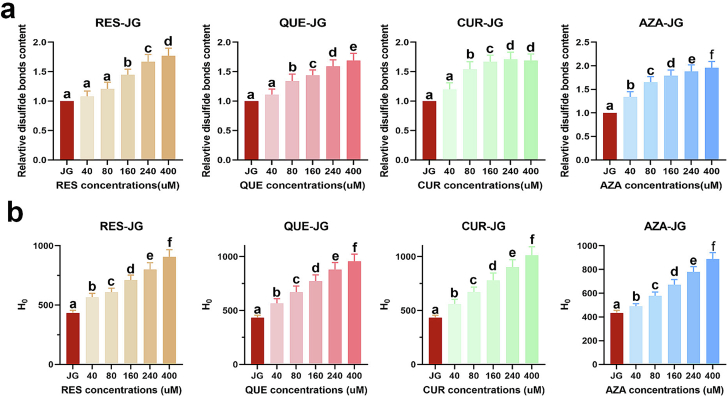
(a) Relative disulfide bonds content changes of JG after combination with RES, QUE, CUR, and AZA. (b) H_0_ changes of JG after combination with RES, QUE, CUR, and AZA. Different letters indicate statistically significant differences at *p* < 0.05.

The disulfide bond in a protein is a covalent linkage formed by sulphur atoms in two cysteine residues (Jiang et al., 2022b). Disulfide bonds play an essential role in protein folding and stability. Alterations in the disulfide bond content significantly impact protein structure. A correlation exists between sulfhydryl group content in proteins and their structural modifications. Sulfhydryl groups can form disulfide bonds, participate in oxidation reactions, catalysis active site reactions, and interacting with reducing agents, thus affecting protein structure and function (Jiang et al., 2022b). Further investigation into the role and regulatory mechanisms of sulfhydryl groups in proteins is of paramount significance for comprehending the relationship between protein structure and function. As depicted in Fig. 4a, the sulfhydryl content of FCTs-JG increased with concentrations compared to the original JG (8%–96%). This result indicates that structural changes in JG expose the intramolecular sulfhydryl group on the surface. Notably, AZA influenced the sulfhydryl content of JG, resulting in an increase ranging from 34% to 96%, indicating that the addition of AZA altered the JG structure and exposed more sulfhydryl groups on the JG surface. A study reported that sulfhydryl groups enable protein formation of disulfide bonds (Jiang et al., 2022b). The formation of disulfide bonds through sulphur atoms between two sulfhydryl groups introduces cross-linking into the protein structure, thereby enhancing stability and rigidity (Cao & Wang, 2022). The stability of the conjugates also increases after binding with FCTs.

### 3.7 H_0_

Protein hydrophobicity plays a significant role in protein folding stability, folding rate, interactions, structure, and function (Jiang et al., 2022b). As shown in Fig. 4b, the tested functional components also demonstrated a concentration-dependent increase in the H_0_ of JG. This phenomenon arises from the hydrophobic nature of the tested FCTs (Jiang et al., 2022c), and their binding to JG enhances its hydrophobicity. RES caused the most hydrophobicity among other components (29.52–133.90%) due to its limited solubility in aqueous solutions. While AZA resulted in the lowest hydrophobicity of JG (13.37–105.09%).

When FCTs bind to proteins, their hydrophobic regions can interact with hydrophobic regions on the surface of the proteins, augmenting the protein's hydrophobicity (Cheng, Zhu, & Liu, 2020). Hydrogen bond donors or acceptor groups in RES, QUE, CUR, and AZA can act as hydrogen bond donors or receptors on the protein surface. The formation of such hydrogen bonds can induce changes in protein conformation, stability, and hydrophobicity (Jiang et al., 2022c). These compounds can interact with hydrophobic residues within JG, facilitating the formation of a hydrophobic core, essential for protein stability and structural intergrity. In addition, RES, QUE, CUR, and AZA can modulate protein conformation by engaging in interactions with the protein. These compounds can modulate the protein's folding state, conformational dynamics, and its binding affinity to other molecules, thereby impacting the protein's hydrophobicity (Cheng et al., 2020).

### 3.8 Antioxidant activities

**Fig. 5:**
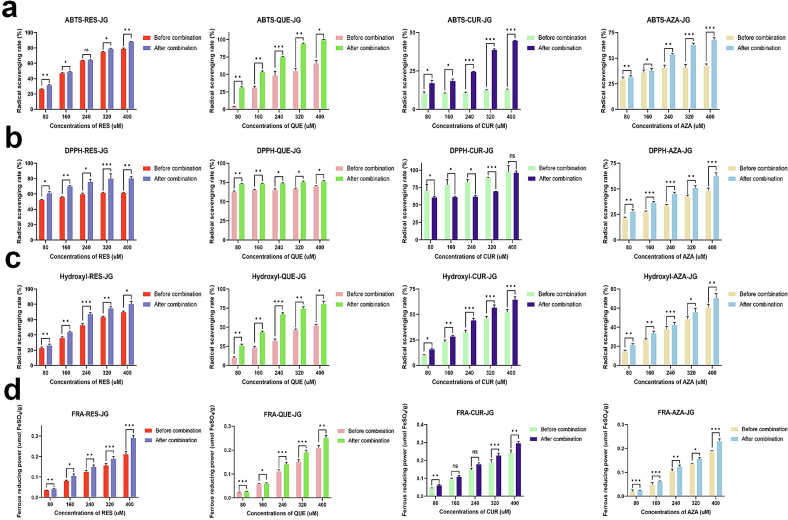
*In vitro* antioxidant activity changes of RES, QUE, CUR, and AZA after combination with JG. (a) ABTS. (b) DPPH. (c) Hydroxyl radical. (d) FRA. *, **, and *** indicate statistically significant differences at p < 0.05, 0.01, and 0.001, respectively.

JG has been recognized for its antioxidant ability (Jiang et al., 2022a). As depicted in Fig. 5, ABTS, DPPH, FRA, and hydroxyl radical scavenging abilities were measured. In most cases, the antioxidant activities of FCTs-JG significantly increased (*p* < 0.05). Concerning ABTS scavenging ability, the FCTs exhibited an amplified ability after combinations. Specifically, QUE-JG exhibited a significant increase (*p* < 0.001) in ABTS radical scavenging ability at a QUE concentration of 240 μM (26.95 ± 2.04%). For RES-JG, with an RES concentration of 320 μM, the most significant improvement was observed, showing an increase of 18.64 ± 1.64% (p < 0.001). However, a significant decrease (p < 0.05) in DPPH scavenging ability was observed in CUR after combination with JG, suggesting that the structures responsible for CUR's DPPH scavenging ability were covered or destroyed. The main reason for the decrease in DPPH free radical scavenging ability is that the interaction between CUR and JG results in structural changes of the two compounds. The binding site of them occurs at the active site of CUR, making it unable to effectively capture DPPH free radicals (von Staszewski, Pilosof, & Jagus, 2011). Furthermore, the combination can generate new compounds with diminished DPPH scavenging ability. The increase in CUR-JG's ABTS scavenging ability indicates that the structure of CUR contributes differently to ABTS and DPPH scavenging abilities. Among all combinations, QUE-JG exhibited the highest increase (34.86 ± 0.67%, p < 0.001) in hydroxyl radical scavenging ability. While CUR-JG exhibited the highest increase in FRA assay (0.04 umol FeSO_4_/g).

The antioxidant activity of compounds is intrinsically linked to their structural characteristics. Phenolic hydroxyl groups in compounds are associated with antioxidant activity (Hoti et al., 2022). These hydroxyl groups effectively trap free radicals, preventing oxidation reactions. Conjugated double bonds can donate electrons to free radicals, stabilizing them and reducing oxidation reactions (Mordi, Ademosun, Ajanaku, Olanrewaju, & Walton, 2020). Compounds endowed with amine groups also manifest antioxidant activity through providing hydrogen atoms that reduce free radical activity (Jiang et al., 2020). Additionally, carboxylic acid groups can provide hydrogen atoms, counteracting the effects of free radicals. Polyphenol compounds excel in trapping multiple free radicals, effectively resisting oxidation. The antioxidant activity of compounds is also modulated by other factors, such as molecular size, solubility, and stability (Mordi et al., 2020). RES's molecules feature multiple phenolic hydroxyl groups (OH), which are critical structural features contributing to its antioxidant activity (Ren et al., 2022). The phenolic hydroxyl group can capture and neutralize free radicals, thus curbing oxidation reactions. The polyphenol hydroxyl group in RES enhances its ability to neutralize free radicals and improves its antioxidant activity. RES, QUE, and CUR molecules contain double-bond structures contributing to their antioxidant activity. Double bonds can trap free radicals through electron transfer reactions, reducing occurrence of oxidation reactions (Ren et al., 2022). The double-bond configuration in RES amplifies its reactivity towards free radicals and blostering its antioxidant activity. The stable molecular structure of RES allows it to maintain antioxidant activity for extended periods in the body. CUR's aromatic ring structure is another key feature associated with its antioxidant activity (Purushothaman et al., 2022). The π electron within the aromatic ring structure can capture and neutralize free radicals, thus mitigating oxidation reactions (Ren et al., 2022). The aromatic ring structure of CUR enhances its reactivity to free radicals and improves its antioxidant activity. The 3-hydroxyl group of the QUE molecule also plays a vital role in its antioxidant activity (Ronsisvalle et al., 2020). This hydroxyl group increases its reactivity with free radicals, enhancing its antioxidant activity. Moreover, JG also possesses radical scavenging abilities due to its small chain active peptides, as reported in the present study (Jiang et al., 2022a). This implies the possibility of a synergistic effect between FCTs and the inner peptides of JG (Jiang et al., 2019).

Antioxidant capacity is further delineated through its reductive capability, wherein antioxidants donate electrons to eliminate free radicals. A stronger reducing ability also indicates better antioxidant ability (Jiang et al., 2020). Common reducing functional groups include hydroxyl (OH), amino, double bond, triple bond, halogen atom (−X), amine group, and alkane group. RES, QUE, and CUR exhibit reducibility due to their hydroxyl and double bonds (Ronsisvalle et al., 2020). The conjugation with JG enhances the reducing power by incorporating polypeptide amine groups and other structures from JG (Cao & Wang, 2022).

AZA can inhibit the hydroxylation of aromatic compounds induced by active oxygen groups and the peroxidation of arachidonic acid *in vitro*, indicating its antioxidant abilities (Xing, Yang, Zhang, & Gao, 2021). JG is easily dissolved in an alkaline solution. When JG combines with AZA, the hydroxyl and amino groups in the alkaline solution combine with the carboxyl groups in AZA to form basic phenolic hydroxyl or amine groups with antioxidant activity (Hoti et al., 2022). The increase of FCTs-JG antioxidant activity is particularly evident with increasing FCTs concentration (Fig. 5). This result is because of the intrinsic antioxidant activity of AZA itself and its protective effect on JG active sites to avoid radicals and other oxidation materials' oxidative effect on JG. The above results demonstrate that the interaction between FCTs and JG enhances the antioxidant activity of the mixture (Jiang et al., 2022a).

### 3.9 Stability during storage

**Fig. 6:**
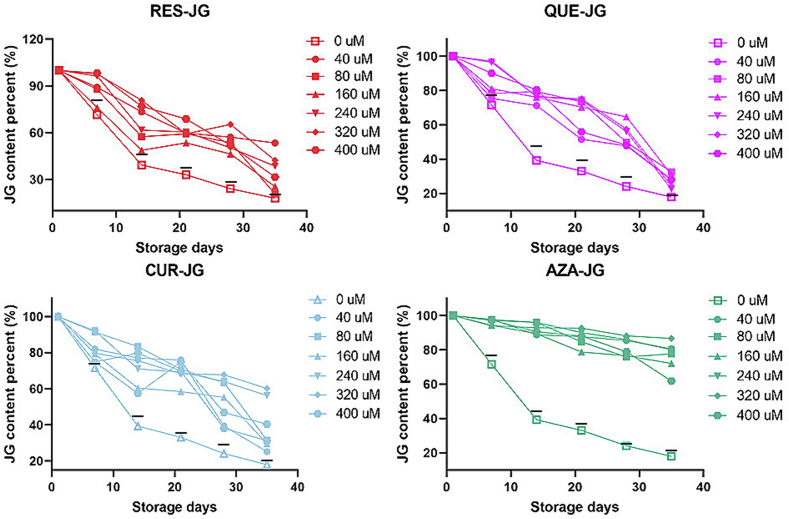
Storage stability of JG changes after combination with RES, QUE, CUR, and AZA.

The stability of a protein is defined by its capacity to retain its structure integrity and functionality under specified storge conditions (Alavi, Chen, Wang, & Emam-Djomeh, 2021). As illustrated in Fig. 6, JG content progressively decreased with the extended storage time due to the action of endogenous enzymes, oxidation, and fluctuations in pH level. After five-week period, JG content declined to 18.00 ± 1.46%. The mixture of JG-FCTs decreased significantly as well (the black lines in Fig. 6), but the JG content was higher than JG alone in most cases due to the antibacterial properties of FCTs protecting JG. However, when combined with RES, QUE, CUR, and AZA, the stability of JG significantly improved. Mixing JG with 40 mM of RES (increased by 35.40 ± 2.71%), 80 mM of QUE (increased by 14.60 ± 1.40%), 320 mM of CUR (increased by 42.17 ± 3.42%), and 320 mM of AZA (increased by 68.70 ± 3.69%) significantly improved its stability in different combinations.

The storage stability of proteins can be influenced by numerous factors, including temperature, pH, oxygen concentration, humidity, and other substances (Abdel-Karim & El-Shamy, 2022). FCTs improve the stability of JG due to their antioxidant properties, which inhibit the oxidation reaction of proteins. When exposed to oxygen, proteins are susceptible to oxidative damage, resulting in structure and function loss. Phenols have the ability to trap and neutralize free radicals, thereby reducing the occurrence of oxidation reactions and protecting proteins from oxidative damage (Pateiro et al., 2021). FCTs can interact with the proteins to stabilize their structure through the formation of interaction forces such as hydrogen bonds, ionic bonds, or van der Waals forces. This interaction enhances protein folding stability, minimizes structural changes during storage or processing, and prevents protein aggregation, maintaining them in a monodisperse state. Protein aggregation can lead to inactivity or the formation of toxic aggregates. Phenols can interfere with the protein aggregation process and maintain a stable monomer state (Zhu et al., 2021). Furthermore, phenols can increase protein hydration by forming hydrogen bonds with water molecules. This hydration process helps preserve protein structure and stability (Nawrocki, Karaboga, Sugita, & Feig, 2019). By providing additional hydration shells, phenols decrease the interaction between proteins and water molecules in the surrounding environment, ultimately reducing protein surface exposure and the propensity to aggregate (Wang et al., 2022). Among the four combinations tested, AZA maintained the highest degree JG stability. This finding is mainly ascribed to the fact that AZA is a dibasic acid that regulates the pH of the environment to maintain protein stability (Xing et al., 2021). Within an optimal pH range, AZA can neutralize pH and prevent structural changes or degradation of proteins under acidic or alkaline conditions. AZA has multiple carboxyl functional groups that can form coordination bonds with metal ions (Hoti et al., 2022). Through metal ion binding, AZA stabilizes the structure of proteins and inhibits oxidation or other detrimental effects caused by metal ions. AZA possesses antioxidant properties that neutralize free radicals, peroxides, and other oxidants (Hoti et al., 2022). Free radicals and oxidants are primary contributors to protein oxidation and degradation. AZA improves protein stability by reducing oxidation reactions. Additionally, AZA exhibits favourable water solubility and can form hydrogen bonds and hydration compounds with water. These hydration actions aid in maintaining the JG's hydrated state and prevent it from losing stability under dry or insufficient water conditions (Cheng et al., 2020). In addition, the antibacterial action of AZA also contributes to the stability of JG.

## 4 Conclusion

This study delves into the stability and antioxidant activity of JG and FCTs (namely RES, QUE, CUR, and AZA), both individually and in combination. The interactions between JG and FCTs were determined to be driven by electrostatic interaction, hydrophobic forces, van der Waals forces, and hydrogen bonding. Notably, combinations resulted in significant changes in the secondary structures of JG. The characteristic peaks of JG in UV, FT-IR, CD, and FL appeared noteworthy changes (red shifts or blue shifts). Additionally, there was an increase in sulfhydryl content and hydrophobicity of JG. The storage stability of JG demonstrated improvement after interacting with FCTs, accompanied by enhanced antioxidant activities. Among all tested combinations, AZA (320 mM)-JG showed the highest stability. QUE (240 and 320 mM)-JG exhibited the most substantial improvement in antioxidant activity.

The interactions between FCTs and JG unveil the potential to enhance the nutritional value of food ingredients, endowing food-specific health benefits tailored to dietary needs. Furthermore, these interactions contribute to enhanced stability during food processing and storage. This preliminary research offers valuable insights into the intricate interplay between FCTs and JG, serving as a reference for future investigations on the synergy between functional ingredients and their applications in functional foods, particularly in terms of stability and functional improvement. Future experiments should further explore the binding mechanism, *in vitro* and *in vivo* digestion stability as well as functional properties of JG-FCTs combinations.

## CRediT authorship contribution statement

**Yunsong Jiang:** Writing – review & editing, Writing – original draft, Software, Methodology, Investigation, Formal analysis, Conceptualization. **Yuxin Qin:** Investigation. **Jayani Chandrapala:** Writing – review & editing. **Mahsa Majzoobi:** Writing – review & editing. **Charles Brennan:** Writing – review & editing. **Jinyuan Sun:** Writing – review & editing, Supervision, Project administration, Funding acquisition. **Xin-An Zeng:** Writing – review & editing. **Baoguo Sun:** Writing – review & editing.

## Declaration of competing interest

All the authors disclose that there are no financial and personal conflict interests that could have appeared to influence the work reported in this paper.

## Data Availability

Data will be made available on request.

## Appendix A Supplementary data

Supplementary material Image 1
