# Supplementary material for: Investigation of interactions between Jiuzao glutelin with resveratrol, quercetin, curcumin, and azelaic and potential improvement on physicochemical properties and antioxidant activities
Source: Food Chem X. 2024 Apr 10;22:101378. doi: 10.1016/j.fochx.2024.101378 (PMC11043818; doi:10.1016/j.fochx.2024.101378)
Supplement: Supplementary file 1 — Supplementary material [file mmc1.docx]

**Supplementary Materials**

**Investigation of interactions between Jiuzao glutelin with resveratrol, quercetin, curcumin, and azelaic and potential improvement on physicochemical properties and antioxidant activities**

Yunsong Jiang^a,b,c^, Yuxin Qin^c^, Jayani Chandrapala^c^, Mahsa Majzoobi^c^, Charles Brennan^c^, Jinyuan Sun^a*^, Xin-an Zeng^b^, Baoguo Sun^a^

^a^Key Laboratory of Geriatric Nutrition and Health, Beijing Technology and Business University, Ministry of Education, 100048, People’s Republic of China

^b^School of Food Science and Engineering, South China University of Technology, Guangzhou, 510641, People’s Republic of China

^c^Biosciences and Food Technology, RMIT University, Bundoora West Campus, Plenty Road, Melbourne, VIC, 3083 Australia

**Table S1**. Quenching constants and thermodynamic parameters of the interaction between RES, QUE, CUR, and AZA with JG.

|  | T(℃) | K_sv_(×10^3^M^-1^) | K_q_(×10^12^M^-1^ S^-1^) | K_A_(×10^4^M^-1^) | n | △H(KJ/mol) | △G(KJ/mol) | △S(J/mol/K) |
| --- | --- | --- | --- | --- | --- | --- | --- | --- |
|  | 30 | 0.3 | 0.03 | 0.00023 | 0.25 | 237.09 | 9.30 | 751.39 |
| RES | 40 | 0.50 | 0.05 | 0.00012 | 4.21 | 237.09 | 1.78 | 751.39 |
|  | 50 | 0.20 | 0.02 | 0.00038 | 0.26 | 237.09 | 5.73 | 751.39 |
|  | 30 | 0.40 | 0.04 | 0.00030 | 1.26 | 160.15 | -3.15 | 0.54 |
| QUE | 40 | 0.80 | 0.08 | 0.015 | 0.63 | 160.15 | -8.53 | 0.54 |
|  | 50 | 0.50 | 0.05 | 0.0035 | 0.47 | 160.15 | -13.92 | 0.54 |
|  | 30 | 2.80 | 0.28 | 14.19 | 1.43 | -356.74 | -29.82 | -1.08 |
| CUR | 40 | 1.60 | 0.16 | 14.12 | 0.84 | -356.74 | -19.03 | -1.08 |
|  | 50 | 1.80 | 0.18 | 0.0022 | 0.35 | -356.74 | -8.25 | -1.08 |
|  | 30 | 2.20 | 0.22 | 13.43 | 1.43 | -74.70 | -30.29 | -0.15 |
| AZA | 40 | 2.40 | 0.24 | 9.65 | 1.35 | -74.70 | -28.83 | -0.15 |
|  | 50 | 2.00 | 0.20 | 2.11 | 1.18 | -74.70 | -27.36 | -0.15 |
